# ABO blood group is a predictor of survival in patients with laryngeal cancer

**DOI:** 10.1186/s40880-016-0152-9

**Published:** 2016-10-13

**Authors:** Ting Jin, Pei-Jing Li, Xiao-Zhong Chen, Wei-Han Hu

**Affiliations:** 1 Key Laboratory of Radiation Oncology in Zhejiang Province, Hangzhou, 310022 Zhejiang P. R. China; 2 Department of Radiation Oncology, Zhejiang Cancer Hospital, 38 Guang Ji Road, Hangzhou, 310022 Zhejiang P. R. China; 3 State Key Laboratory of Oncology in South China, Department of Radiation Oncology, Collaborative Innovation Center of Cancer Medicine, Sun Yat-sen University Cancer Center, No. 651 Dongfeng Road East, Guangzhou, 510060 Guangdong P. R. China

**Keywords:** The ABO blood group, Laryngeal cancer, Prognosis, Survival

## Abstract

**Background:**

Whether the ABO blood group is associated with the survival of patients with laryngeal cancer remains unknown. The purpose of this study was to investigate the association between the ABO blood group and clinicopathologic characteristics of patients with laryngeal cancer and assess whether the ABO blood group was associated with prognosis.

**Methods:**

We analyzed the records of 1260 patients with laryngeal cancer who underwent curative treatment at Sun Yat-sen University Cancer Center between January 1993 and December 2009. The Chi-square test was used to assess the relationship between the ABO blood group and clinicopathologic characteristics. The Kaplan–Meier method was used to estimate 3-, 5-, and 10-year overall survival (OS) rates. The Cox proportional hazards model was used in univariate and multivariate analyses of OS.

**Results:**

No significant association was found between the ABO blood group and clinicopathologic characteristics except for primary tumor site. The median OS for patients with blood groups A, B, AB, and O were 87.0, 80.0, 90.0, and 72.5 months, respectively. The 3-, 5-, and 10-year OS rates were 82.4%, 76.0%, and 67.5% for patients with blood group A; 77.4%, 69.8%, and 58.4% for patients with blood group B; 82.2%, 73.1%, and 65.6% for patients with blood group AB; and 71.7%, 66.4%, and 55.5% for patients with blood group O, respectively. Univariate and multivariate analyses showed that the ABO blood group had significant effects on prognosis in patients with laryngeal cancer.

**Conclusions:**

The ABO blood group is associated with survival in patients with laryngeal cancer. Patients with blood group O had significantly shorter OS than patients with other ABO blood groups.

## Background

In the United States, it is estimated that approximately 53,640 new cases of oral cavity, pharyngeal, and laryngeal cancers were diagnosed in 2013. During the same period, an estimated 11,520 deaths from head and neck cancers occurred [1]. Approximately 20% of head and neck tumors originate in the larynx [1]. Laryngeal cancer can be largely prevented by controlling behavioral risk factors, such as alcohol consumption and cigarette smoking. Human papillomavirus infection was also found to be significantly associated with the risk of laryngeal cancer [2]. Importantly, some patients without strong risk factors present with particularly aggressive laryngeal carcinoma. Such cases mandate the investigation of other possible predisposing factors, such as the ABO blood group [3].

Several previous studies, which, taken together, included a relatively small number of patients, examined the relationship between the ABO blood group and the incidence of laryngeal cancer. In 1992, in a study of people living in southern Poland, Konieczna et al. [4] found that the blood group A2B was present significantly more frequently in 153 patients with epiglottic cancer compared with 22,422 healthy individuals; however, the difference was not significant with respect to 3- and 5-year survival rates. In 1995, in another study of people living in Poland, Pyd et al. [5] reported that the blood group A2 was present significantly more frequently in a group of patients with glottic cancer compared with supraglottic and subglottic cancer and that the blood group A1B was present significantly more frequently in a group of patients with hypopharyngeal cancer compared with laryngeal caner. Conversely, in 2000, Nowinska et al. [6] retrospectively studied yet another population living in southern Poland and found that differences among distinct ABO blood groups were not significant between 205 laryngeal cancer patients and 5168 healthy individuals. Few studies have examined the effect of the ABO blood group on the survival of patients with laryngeal cancer. Adam et al. [3] reported no association between the ABO blood group and 5 year survival and mortality in 143 laryngeal cancer patients.

Whether the ABO blood group is associated with the survival of patients with laryngeal cancer remains unknown. Therefore, we retrospectively studied a consecutive cohort of patients in China who presented with locoregional laryngeal cancer and underwent curative treatment. The purpose of this study was to elucidate the association between the ABO blood group and the clinicopathologic characteristics of patients with laryngeal cancer and determine whether a specific ABO blood group is an independent predictor of prognosis.

## Methods

### Ethics, consent, and permissions

This study was approved by the Human Ethics Approval Committee at Sun Yat-sen University Cancer Center (registration number: B2011-06-15); requirement to obtain informed consent was waived.

### Patients

We reviewed the records of patients treated at Sun Yat-sen University Cancer Center between January 1993 and December 2009 and found 1477 patients with primary squamous cell carcinoma of the larynx. The patient selection criteria were as follows: (1) each patient had complete clinicopathologic data, including age, sex, stage, smoking index, alcohol consumption, histologic differentiation, the ABO blood group, and treatment status; (2) diseases were restaged according to the 2002 Union for International Cancer Control (UICC) cancer staging system, and no patient had distant metastasis at the time of initial staging; and (3) patients were initially treated with curative intent by one or a combination of surgery, radiation therapy, and chemotherapy. The ABO blood group (A, B, O, or AB) was determined using mouse-derived monoclonal antibodies (Ortho Bioclones Anti-A, B, and O; Ortho Diagnostic Systems Inc., Raritan, NJ, USA). After cessation of treatment, each patient was followed up every 3 months at the clinic or by telephone contact for an interview. The last follow-up was December 31, 2013.

### Treatment

Surgical methods included total laryngectomy, partial laryngectomy, and chordectomy for primary tumors. In the surgical treatment of the neck, modified radical neck dissection (MRND) and selective neck dissection (SND) were used. Cisplatin-based regimen was used for neoadjuvant chemotherapy, adjuvant chemotherapy, and concurrent chemoradiotherapy. Radiotherapy was delivered employing ^60^Co units or 6-MV linear accelerator. Total radiation dose in the area of clinical target volume ranged from 50 to 82 Gy (median, 60 Gy).

### Statistical analyses

Overall survival (OS) was calculated from the date of diagnosis to the date of death or the last follow-up. The relationship between the ABO blood group and clinicopathologic variables was assessed by the Chi-square test. For univariate analysis, OS was estimated using the Kaplan–Meier method, and the statistical significance of differences between curves was tested using the log-rank test. To determine independent prognostic factors, variables found to have a significant association with OS (*P* < 0.05) on univariate analyses were included in the multivariate models. *P* values less than 0.05 were considered statistically significant. All statistical analyses were performed using the Statistical Package for the Social Sciences software (SPSS version 21.0; SPSS Inc., Chicago, IL, USA).

## Results

### Overall study population

Baseline clinicopathologic characteristics of the whole study population are listed in Table 1. In total, 1260 patients were identified for our study; of these, 1226 were men, and 34 were women. For the whole cohort of patients, the median age at diagnosis was 61 years (range, 22–93 years). A total of 747 patients had UICC stage I–II disease, and 513 had UICC stage III–IV disease. Eight hundred eighty-five patients had glottic cancer, 331 had supraglottic cancer, and 44 had subglottic cancer. Additionally, 808 patients had never consumed alcohol, whereas 452 patients had. Three hundred forty-seven patients (27.5%) had blood group A, 332 had blood group B (26.4%), 101 had blood group AB (8.0%), and 480 had blood group O (38.1%). No significant association was found between the ABO blood group and sex, age, smoking index, alcohol consumption, tumor differentiation, T category, N category, and UICC TNM stage (Table 1).

**Table 1:** Baseline characteristics of 1260 laryngeal cancer patients divided by different ABO blood groups

Characteristic	Total	ABO blood group [cases (%)]				χ^2^	*P*
		A (*n* = 347)	B (*n* = 332)	AB (*n* = 101)	O (*n* = 480)		
Sex						1.576	0.665
Men	1226	338 (27.6)	320 (26.1)	99 (8.1)	469 (38.2)		
Women	34	9 (26.5)	12 (35.3)	2 (5.9)	11 (32.3)		
Age (years)^a^						2.122	0.547
≤61	661	176 (26.6)	168 (25.4)	53 (8.0)	264 (40.0)		
>61	599	171 (28.5)	164 (27.4)	48 (8.0)	216 (36.1)		
Smoking index^b^						4.010	0.260
≤600	659	191 (29.0)	178 (27.0)	45 (6.8)	245 (37.2)		
>600	601	156 (26.0)	154 (25.6)	56 (9.3)	235 (39.1)		
Alcohol consumption						4.899	0.179
No	808	219 (27.1)	226 (28.0)	69 (8.5)	294 (36.4)		
Yes	452	128 (28.3)	106 (23.5)	32 (7.1)	186 (41.1)		
Tumor differentiation						3.452	0.750
Well	600	170 (28.4)	149 (24.8)	54 (9.0)	227 (37.8)		
Moderate	481	132 (27.4)	134 (27.9)	32 (6.7)	183 (38.0)		
Poor	179	45 (25.1)	49 (27.4)	15 (8.4)	70 (39.1)		
Primary site						16.755	0.010
Glottic	885	244 (27.6)	242 (27.3)	79 (8.9)	320 (36.2)		
Supraglottic	331	83 (25.1)	82 (24.8)	22 (6.6)	144 (43.5)		
Subglottic	44	20 (45.5)	8 (18.2)	0 (0)	16 (36.3)		
T category						7.212	0.615
T1	434	119 (27.4)	123 (28.4)	31 (7.1)	161 (37.1)		
T2	382	115 (30.1)	94 (24.6)	31 (8.1)	142 (37.2)		
T3	244	58 (23.8)	65 (26.6)	26 (10.7)	95 (38.9)		
T4	200	55 (27.5)	50 (25.0)	13 (6.5)	82 (41.0)		
N category						2.319	0.509
N0	1021	289 (28.3)	269 (26.3)	83 (8.1)	380 (37.2)		
N+	239	58 (24.3)	63 (26.4)	18 (7.5)	100 (41.8)		
UICC stage						9.077	0.430
I	421	118 (28.0)	118 (28.0)	29 (6.9)	156 (37.1)		
II	326	101 (31.0)	77 (23.6)	27 (8.3)	121 (37.1)		
III	242	54 (22.3)	66 (27.3)	26 (10.7)	96 (39.7)		
IV	271	74 (27.3)	71 (26.2)	19 (7.0)	107 (39.5)		

*UICC* Union for International Cancer Control

^a^Patients were divided into two groups according to the median age

^b^Smoking index was defined as the number of cigarettes smoked per day × the total smoking duration (years)

Of the 1260 patients identified, 795 underwent radical resection alone, 68 received definitive radiation alone, 14 received definitive concurrent chemoradiotherapy, 298 underwent surgery plus postoperative radiotherapy, 23 underwent surgery plus adjuvant chemotherapy, 11 underwent surgery plus adjuvant concurrent chemoradiotherapy, 7 received radiotherapy before surgery, 4 received neoadjuvant chemotherapy plus surgery, 24 received neoadjuvant chemotherapy plus definitive concurrent chemoradiotherapy, 1 received concurrent chemoradiotherapy before surgery, 5 underwent surgery plus postoperative radiotherapy and adjuvant chemotherapy, 6 received neoadjuvant chemotherapy plus surgery and postoperative radiotherapy, 1 received neoadjuvant chemotherapy plus surgery and adjuvant chemotherapy, 2 received neoadjuvant chemotherapy plus surgery and adjuvant concurrent chemoradiotherapy, and 1 received neoadjuvant chemotherapy plus concurrent chemoradiotherapy and surgery.

### Survival analyses by the ABO blood group

**Fig. 1:**
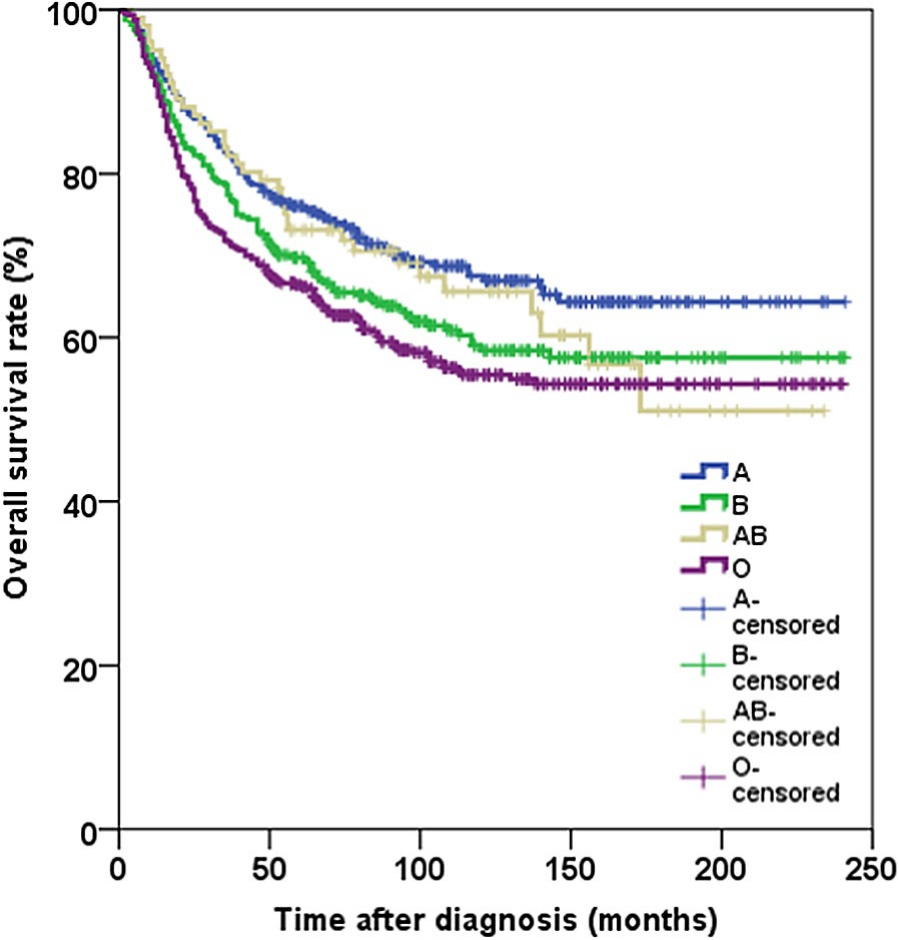
Overall survival curves for 1260 laryngeal cancer patients stratified by the ABO blood group. The 3-, 5-, and 10-year OS rates were 82.4%, 76.0%, and 67.5% for patients with blood group A; 77.4%, 69.8%, and 58.4% for patients with blood group B; 82.2%, 73.1%, and 65.6% for patients with blood group AB; and 71.7%, 66.4%, and 55.5% for patients with blood group O, respectively (*P* = 0.009)

For the entire patient group, the median OS was 80 months; the 3-, 5-, and 10-year OS rates were 77.5%, 70.5%, and 60.4%, respectively. The 3-, 5-, and 10-year OS rates were 82.4%, 76.0%, and 67.5% for patients with blood group A; 77.4%, 69.8%, and 58.4% for patients with blood group B; 82.2%, 73.1%, and 65.6% for patients with blood group AB; and 71.7%, 66.4%, and 55.5% for patients with blood group O, respectively. The median OS for patients with blood groups A, B, AB, and O was 87.0, 80.0, 90.0, and 72.5 months, respectively. We found a significant difference in OS between different ABO blood groups (*P* = 0.009; Fig. 1). Furthermore, for the entire cohort, we analyzed the association of the ABO blood group with OS rates. For patients with laryngeal cancer, univariate analyses indicated a significant association between OS rates and sex, age, smoking index, alcohol consumption, tumor differentiation, the ABO blood group, primary tumor site, T category, N category, and UICC TNM stage (Table 2).

**Table 2:** Univariate prognostic analysis for overall survival (OS) rates of 1260 patients with laryngeal cancer

Variable	No. of patients	OS rate (%)		*χ* ^*2*^	P
		5-year	10-year		
Sex				6.079	0.014
Men	1226	70.0	59.7		
Women	34	85.3	85.3		
Age (years)^a^				35.892	<0.001
≤61	661	76.0	68.7		
>61	599	64.4	51.5		
Smoking index^b^				8.322	0.004
≤600	659	73.2	64.1		
>600	601	67.4	56.2		
Alcohol consumption				4.933	0.026
No	808	72.3	62.5		
Yes	452	67.2	56.6		
Differentiation				27.775	<0.001
Well	600	76.8	65.7		
Moderate	481	68.5	58.4		
Poor	179	54.6	48.1		
Primary site				31.102	<0.001
Glottic	885	74.8	64.8		
Supraglottic	331	58.6	48.7		
Subglottic	44	72.7	56.2		
Blood group				11.637	0.009
A	347	76.0	67.5		
B	332	69.8	58.4		
AB	101	73.1	65.6		
O	480	66.4	55.5		
T category				101.754	<0.001
T1	434	84.2	72.0		
T2	382	74.3	63.0		
T3	244	59.4	51.4		
T4	200	46.8	41.1		
N category				119.722	<0.001
N0	1021	76.9	66.1		
N+	239	43.0	35.4		
UICC stage				127.473	<0.001
I	421	85.4	73.5		
II	326	77.2	65.3		
III	242	62.4	52.6		
IV	271	46.3	41.4		

*UICC* Union for International Cancer Control

^a^Patients were divided into two groups according to the median age

^b^Smoking index was defined as the number of cigarettes smoked per day × the total smoking duration (years)

Multivariate analysis showed that age, alcohol consumption, tumor differentiation, the ABO blood group, T category, and N category were independent prognostic factors (Table 3).

**Table 3:** Multivariate analysis of OS with the Cox proportional hazards model

Variable	Hazard ratio	95% CI	*P*
Alcohol consumption	1.218	1.009–1.470	0.040
Tumor differentiation	1.201	1.057–1.365	0.005
Primary site	0.889	0.738–1.071	0.215
T category	1.334	1.213–1.468	<0.001
ABO blood group	1.101	1.023–1.184	0.010
Sex	0.425	0.175–1.034	0.059
Age	1.877	1.558–2.260	<0.001
Smoking index	1.122	0.934–1.349	0.220
N category	2.203	1.749–2.774	<0.001

*CI* confidence interval

## Discussion

In our study of 1260 patients with laryngeal cancer, we investigated the association between the ABO blood group and clinicopathologic characteristics and patient prognosis. We found no significant association between clinicopathologic characteristics and the ABO blood group. Univariate and multivariate analyses showed that the ABO blood group was significantly associated with the prognosis of patients with laryngeal cancer.

There are more than 100 recognized blood group systems composed of more than 500 antigens [7], of which the ABO blood group is considered the most important [8]. The ABO blood group is determined by the presence of A or B blood group antigens on the surface of red blood cells, which consist of proteins and carbohydrates attached to lipids or proteins. Red blood cell antigens have various functions, such as membrane structural integrity, transportation of molecules through membranes, and adhesion [9]. Along with their expression on red blood cells, ABO antigens are highly expressed on human tissues and most epithelial and endothelial cells [10, 11]. Since the first report by Aird et al. [12] that showed an association between blood group A and gastric cancer, the relationship between the ABO blood group and the risk, incidence, and clinicopathologic characteristics of human tumors has been suspected. Moreover, many studies have suggested a possible relationship between ABO blood group antigens and progression of human tumors [13–18]. Several plausible mechanisms, such as inflammation, immune-surveillance of malignant cells, and membrane signaling, have been proposed to explain this observed association between the ABO blood group and cancer risk [19]. Whereas the relationship between the ABO blood group and the incidence of laryngeal cancer remains unclear [4–6], the association between the ABO blood group and the risk of pancreatic cancer has been reported for over 40 years. Compared with people with blood group O, people with non-O blood groups have an adjusted hazard ratio (HR) for pancreatic cancer of 1.44 (95% confidence interval [CI] 1.14–1.82) [19]. Many studies have been conducted to examine the underlying mechanism of this relationship. For example, the multinational Pancreatic Cancer Cohort Consortium identified pancreatic cancer susceptibility loci in the ABO gene [20]. In this study, 1896 patients with pancreatic cancer and 1939 controls were genotyped, and a significant association was reported with rs505922, a single nucleotide polymorphism (SNP) that maps to the first intron of the ABO gene. The ABO SNP rs505922 is in strong linkage disequilibrium with O/non-O blood group alleles, indicating that people with non-O blood groups are at increased risk for developing pancreatic cancer [19, 21, 22]. In addition, two recent genome-wide association studies identified variants in ABO (rs505922), 1q32.1 (rs3790844), 13q22.1 (rs9543325), and 5p15.3 (rs401681) that were associated with a modestly increased risk of pancreatic cancer [23]. Two other studies suggested that the association between A blood group and increased risk of pancreatic cancer is due mainly to the A1 allele, thus indicating a direct connection between ABO glycosyltransferase activity and increased risk of this disease [24, 25]. Risch et al. [26] reported that the increased risk of pancreatic cancer among people with non-O blood groups was even higher if they were also seropositive for CagA-negative Helicobacter pylori (odds ratio: 2.78; 95% CI 1.49–5.20). Recently, Hofmann et al. [27] reported that healthy controls displayed significantly higher isoagglutinin titers and higher rate of binding to Tn and T antigen compared with patients with pancreatic ductal adenocarcinoma. Because Hofmann et al. [27] did not find an association between isoagglutinin titers and clinical parameters (such as OS and tumor stage), they assumed that isoagglutinins are important during tumorigenesis but not during actual tumor growth.

Our study showed, by both univariate and multivariate analyses, that the ABO blood group was an independent prognostic factor for patients with laryngeal cancer. Compared with patients with other ABO blood groups, patients with blood group O had significantly shorter OS.

There are only a few comparable studies in laryngeal cancer or in other types of head and neck cancer. A study by Ouyang et al. [28] indicated that nasopharyngeal carcinoma (NPC) patients with blood group A had significantly lower OS rate (adjusted HR = 1.49 [95% CI 1.03–2.17]) and distant metastasis-free survival rate (HR = 1.68 [95% CI 1.13–2.51]) than patients with non-A blood groups (B, AB, and O). In the subgroup analyses, they found that the increased risks associated with blood group A were restricted to men. Sheng et al. [17] conducted a case–control study and found that male NPC patients with blood group A had a significantly higher rate of distant metastasis than male patients with non-A blood groups (6.8% vs. 3.5%, *P* = 0.027), which directly supports the poorer prognosis of men with blood group A. A possible reason for the different results between laryngeal cancer and NPC is that NPC has a distinct epidemiology, etiology, and clinical manifestation compared with other head and neck cancers, including laryngeal cancer [29, 30].

Our findings are similar to those of previous studies in non-muscle invasive bladder urothelial carcinoma and locoregional esophageal squamous cell carcinoma (ESCC). Klatte et al. [31] found that non-muscle invasive bladder urothelial carcinoma patients with blood group O had higher recurrence and progression rates than patients with blood group A (*P* = 0.015 and 0.031, respectively) or blood group B (*P* = 0.004 and 0.075, respectively). In a subgroup analysis of 321 patients with ESCC who had ever smoked, Sun et al. [32] found that patients with blood group B/O had lower OS rate than patients with blood group A/AB (*P* = 0.024). For ESCC patients who had ever smoked, multivariate analysis showed an unfavorable and independent effect of blood group B/O on survival (*P* = 0.011). Our findings, however, are not in line with the findings of previous studies on pancreatic cancer, renal cell carcinoma, and curatively resected non–small cell lung cancer (NSCLC). For example, Engin et al. [33] and Rahbari et al. [34] found that pancreatic cancer patients with blood group O had a significantly longer survival than patients with non-O blood groups, regardless of prognostic factors. Kaffenberger et al. [35] showed that, in patients with renal cell carcinoma who underwent nephrectomy or partial nephrectomy, non-O blood groups were significantly associated with decreased OS (HR = 1.68, 95% CI 1.18–2.39, *P* = 0.004). Li et al. [36] showed that NSCLC patients with blood group O or B had significantly longer OS, disease-free survival, and local recurrence-free survival than NSCLC patients with blood group A or AB.

The results of studies evaluating the prognostic value of the ABO blood group in various cancers are quite conflicting. There are several possible explanations for the heterogeneity of findings across the studies. First, many studies were performed without the understanding that ABO frequencies can vary widely in populations assumed to be ethnically homogeneous; therefore, they included a limited number of patients and inappropriate control groups. Second, many studies were retrospective. Third, many recent studies that determined the association between the ABO blood group and the occurrence of malignant neoplastic disease are still preliminary or controversial, frequently not supported by strong statistical data. Underlying mechanisms still need to be explored or confirmed.

In our study, the distribution of the ABO blood group was similar to that in the Zhejiang [17] and Guangdong populations [32], with blood group O having the highest percentage (38.1%) and blood group AB having the lowest percentage (8.0%). It is still unclear why ABO blood groups affect the survival of patients with laryngeal cancer. Since no significant differences in the basic characteristics of patients with different ABO blood groups were observed, it is difficult to explain the effect of the ABO blood group. However, underlying molecular and pathogenic differences may play important roles in the effect of the ABO blood group on survival.

Our study had several limitations. First, it was conducted at a single center. Second, to detect an association between the ABO blood group and survival of patients with laryngeal cancer, we selected only the patients with locoregional disease that underwent curative treatment. Therefore, metastatic cases were excluded and not discussed. Third, despite the fact that the patients enrolled in this study mostly came from Guangdong province, some of them came from other areas in China. Fourth, worldwide, the general sex ratio (men:women) of laryngeal cancer is 8–10:1, but in our study, the ratio was 36.1:1. These limitations weaken the applicability of our data.

In conclusion, this study provides evidence of an association between the ABO blood group and survival of patients with laryngeal cancer: patients with blood group O had lower OS rate than patients with non-O blood groups. Further basic research on tumor genetic or biological differences associated with the ABO blood group is needed.
